# Chronic Meningitis, with Partial Paralysis

**Published:** 1887-07

**Authors:** N. S. Davis

**Affiliations:** Professor of Principles and Practice of Medicine in the Chicago Medical College, and Physician to Mercy Hospital


### CHRONIC MENINGITIS, WITH PARTIAL PARALYSIS.

A CLINICAL LECTURE BY N. S. DAVIS, JR., A.M., M.D., PROFESSOR OF PRINCIPLES AND PRACTICE OF MEDICINE IN THE CHICAGO MEDICAL COLLEGE, AND PHYSICIAN TO MERCY HOSPITAL.

THE history and appearance of the patient before you, and whose symptoms are somewhat unique, may thus be described. He is now about sixty years of age, and enjoyed good health until about five years ago. He was, however, always of a very nervous temperament. Six years ago he was involved in business troubles, which caused him much anxiety and worry. He began also to complain of the feeling of a band drawn tightly around his head which, as he expressed it, he wished he could cut. At the same time he began to grow timid, not being willing to go out or to be left alone. By degrees he became more and more dizzy, or rather uncertain, while walking. He felt that the floor was rising constantly to meet his feet. As these symptoms developed, his head began, also, to assume a retracted position, being drawn backward and held by the tensely contracted muscles of the neck. This symptom remained constant, but varied in intensity. The retraction seemed greatest while he was walking, especially while going down stairs.

About one year ago speech became imperfect, and swallowing became difficult. His eyes also began to fail, so that vision was imperfect.

He entered the hospital June 6th, 1885. His appearance has not materially changed since then. As you see, he now appears very feeble and emaciated. His head is constantly drawn backward, and as I attempt to draw it forward its rigidity in this position is manifest. His countenance appears wan, and to the observer the thought is constantly suggested that he is about to weep. The corners of his mouth are drawn down and his lips kept almost constantly apart. The muscles upon the right side of his face are relaxed, and appear as they do in one affected, to a moderate extent, by hemiplegia. His tongue, you observe, is protruded in a normal manner. Sight is dim, and most so with the right eye. Hearing, on the other hand, is noticeably acute. The sense of touch does not seem to be impaired. In answering questions you may have noticed that his speech is slow, low and thick or muffled. Swallowing is very difficult. As he takes the first teaspoonful of water you will probably see that it passes quite easily down the throat, although this is not uniformly so, but the second and third will be accompanied by much gurgling in the throat, and more will cause regurgitation into the mouth and an overflow through the lips, and distress about the neck. Occasionally, also, when regurgitation occurs, liquids escape in part through the nares. Owing to this inability to swallow well, the saliva is constantly dribbling from his half-open mouth, or is feebly expectorated at times. This condition causes very great annoyance and discomfort. The position of the head we have already noticed. He never during his sickness has complained of pain or tenderness about it. Often, however, the back of the head and neck feel hot.

The heart-beats I find to be fairly strong, but intermitting. There are two or three steady beats and then a flutter, which embraces probably two beats. The respiratory movements are somewhat feeble, and the lungs do not fill well. He does not cough. The inspirating sound is hitching at the apices of the lungs, and you notice that there is slight dullness in this region. He had no trouble in urinating until he entered the hospital. The first day after his arrival he was unable to micturate, and the catheter had to be used. This difficulty has not recurred. The bowels move only by an enema, which is administered every second day. The limbs are emaciated and weak. The muscles seem most feeble and least useful upon the right side. He is able, however, to make all the usual movements, but he cannot stand without support, nor walk without assistance. He never sleeps longer than a few minutes or an hour at a time, and always awakes in a fright and often starts up from his bed. He is exceedingly timid, unwilling to be alone, and fearful that something will happen to him.

Interest in this case centers chiefly around the diagnosis. It is quite evident that we have to deal with a disease of the nervous system which has caused a partial paralysis of the muscles used in articulation and deglutition, and a constant contraction of the muscles of the neck. In considering the diagnosis of diseases of the nervous system, it is convenient always first to locate the lesion, as well as possible, and then to determine its nature. Interference with speech and deglutition may be produced by disease of the brain cortex, its deeper structure, and of the medulla or of the nerve trunks. An involvement of the nerves of speech and deglutition is not likely to take place bilaterally, as in this case, unless the lesion is one that involves their roots within the cranium. The origins of the hypoglossal, glossopharyngeal, pneumogastric, spinal accessory, and facial are close together, and diseases of the meninges, such as tumor and inflammation, have been known to involve a part or all of them simultaneously. Disease of the brain that would involve speech and deglutition invariably, we may say, is accompanied by paralysis of other portions of the body, and only rarely produces symmetrical paresis. Lesions in the medulla may produce very similar symptoms to those we see here. In many ways cases of that somewhat rare disease known as progressive bulbar paralysis, or glosso-labio-laryngeal paralysis, resembles this one. One of the earliest symptoms in progressive bulbar paralysis is difficulty of speech. The tongue is not perfectly under command. Not only is there an inability to use it properly but an atrophy of it. By these changes speech is interfered with and also the act of swallowing; next, usually, the lips are affected by both partial loss of motion and atrophy. The aspect is peculiar and very similar to that of the patient before us. The mouth is constantly about half open, the lower lip drooping, the corners of the mouth being drawn down. The whole countenance presents a lachrymose appearance. Usually saliva dribbles from the mouth. The pharyngeal and laryngeal muscles are atrophied and paretic. In this disease there is degeneration of the nuclei in the medulla, which give origin to the nerves of speech and deglutition. As a result of the involvement of the nuclei we find both loss of motion and atrophy of the muscles. In the case before us, however, there is no atrophy of the muscles of the lips, tongue or soft palate. In progressive bulbar paralysis, where the muscles of the neck are involved, it is in atrophy which causes a constant forward drooping of the head, but in the present case the reverse of this is seen; there is retraction of the head and rigidity of the muscles of the back of the neck. The history of this patient does not correspond closely with that of bulbar paralysis. We may therefore reject this from the possibilities in forming our diagnosis. The other lesions confined to the medulla are acute ones, as, for example, hæmorrhage into the medulla or embolism or thrombosis and inflammation or acute bulbar paralysis. Not only are these diseases sudden in their onset, but acute in their course and usually, at least in the onset, the symptoms are not so strictly local.

We have left to consider the possibility of a tumor or of chronic meningeal inflammation as a cause of the symptoms. Tumors in this region are usually accompanid by intense pain and vomiting. Paralysis of the ocular nerves often is produced but not so often of the muscles of speech and deglutition. The diagnosis of tumors of the medulla is difficult to make, for the symptoms produced by them are quite variable, the most common, however, being those mentioned. Could a chronic inflammation of the meninges cause the symptoms that we find here? This question must be answered in the affirmative, and without doubt this is the most plausible diagnosis of this case. By such inflammation the nerves are involved at their roots, so as to be functionally imperfect. The retraction of the head also suggests inflammatory irritation of the upper part of the cord or cervical nerves. The heat that is noticed at times about the back of the neck and head might be expected under these circumstances. Usually pain is a prominent symptom of meningeal inflammation, and somewhat singularly this patient has never complained of pain. The girdle sensation about the head is the nearest approach to pain of anything of which he has spoken.

As regards the ætiology of inflammation in this region, it is said most frequently to be the result of syphilitic or alcoholic poisoning. Intense and protracted anxiety, injuries, continued fevers, epidemic cerebro spinal meningitis, rarely acute meningitis and suppurative otitis are other causes. There is in this case no history of syphilitic infection or alcoholic excess, or of injury, or fever, or prior meningitis of any kind. There is, however, a history of intense and prolonged mental anxiety and worry.

The prognosis must be an unfavorable one. The case has been of so long standing that if ever there was hope of cure there can be scarcely any now. The treatment has been chiefly palliative, although some drugs have been employed with the hope of modifying the morbid processes, but unavailingly. For this latter purpose the iodides chloride of gold, ergot and physostigma have been used faithfully. Sleep cannot be deepened or prolonged without rendering him stupid with very large doses of opiates or other hypnotics.

CLINIC AFTER AUTOPSY.

*Gentlemen:* You will recall the case of an old gentleman who was shown to the clinical class a month and a half ago, whose symptoms pointed to an involvement of the medulla or bulbar nerves, and in regard to whom we made the probable diagnosis of meningitis about the medulla, and possibly the upper part of the cord.

The patient remained in practically the condition in which he was seen by you from the time he entered the hospital until August 10th, when he began to complain of pain in the bowels and had frequent passages, mostly fluid, with small particles of dry, hard fæces. The pain caused considerable suffering. It was partly allayed by full doses of opiates. After the second day he became very dull; respiration was shallow and there were signs of œdema of the lungs. The right half of the body seemed also less perfectly under control of the will than before. His respiratory movements grew gradually weaker until death occurred. An autopsy was made a few hours later. On opening the cranial cavity the walls of the membranes appeared full of dark blood. The membranes also were distended by an accumulation of serum beneath the pia meter. Adhesions between dura and pia mater were present over the upper surface of the cerebrum near the central line. There were here and there in the pia mater and below it, extending into the sulci, white opaque and firm patches, evidently of inflammatory origin. The pachionian bodies were more numerous than normal, and about them the minute vessels were distended with blood. The dura and pia maters were thickened in places. There were also adhesions between the cerebellum and the cerebrum on the left side. About the upper portion of spinal column and medulla there were close adhesions and bands of new tissue. These had to be dissected away before the brain could be removed. The adhesions were particularly close about the hypoglossal, but involved the neighboring nerve roots to some extent. They were firmest, also, upon the right side. The ventricles were distended slightly with serous fluid. A microscopical examination of medulla revealed no abnormal appearances. There was nothing in the changes in the other organs that threw light upon the early history of the case.

The pericardium, the pericardial cavity and the heart were normal in size, shape and structure. Old and firm pleural adhesions were found over the right lung. On section, the lungs appeared œcleastian, with near the apex a few quite small firm patches of cirrhosis, and with here and there small neighboring patches of emphysema. The left lung was free in its pleural cavity and presented appearances exactly corresponding to those of the right. The areas of firm cirrhosis were, however, more numerous.

The kidneys were small and soft. The capsule slightly adherent. The cortex, especially near the middle, was narrow, pale and grayish yellow.

The liver was normal in size, shape and consistency, but somewhat paler than natural.

Nothing abnormal was observed about the stomach, intestines or bladder.

The autopsy, therefore, shows clearly that the case was one of chronic inflammation of the meninges, chiefly about the medulla and base of the brain.

